# Carboplatin versus cisplatin in combination with etoposide in the first-line treatment of small cell lung cancer: a pooled analysis

**DOI:** 10.1186/s12885-021-09034-6

**Published:** 2021-12-07

**Authors:** Shiyu Jiang, Liling Huang, Hongnan Zhen, Peijie Jin, Jing Wang, Zhihuang Hu

**Affiliations:** 1 Department of Medical Oncology, Fudan University Shanghai Cancer Center; Institute of Thoracic Oncology, Fudan University, 270 Dongan Rd, Shanghai, 200032 China; 2 Department of Medical Oncology, Cancer Hospital, Chinese Academy of Medical Sciences & Peking Union Medical College, Beijing, 100021 China; 3 Department of Radiation Oncology, Peking Union Medical College Hospital, Chinese Academy of Medical Sciences and Peking Union Medical College, Beijing, China; 4 Philips Research, Shanghai, 200032 China; 5 Department of Anesthesiology, Fudan University Shanghai Cancer Center, Shanghai, 200032 China; 6 Department of Oncology, Shanghai Medical College, Fudan University, Shanghai, China

**Keywords:** Adverse events, Extensive-stage, Small cell lung cancer, Treatment

## Abstract

**Background:**

Extensive-stage small cell lung cancer (ES-SCLC) is an aggressive disease with poor survival, and platinum-etoposide chemotherapy is indicated as the mainstay of treatment. In this study, we compared the efficacy and safety between the cisplatin plus etoposide (EP) and carboplatin plus etoposide (EC) regimens.

**Methods:**

A total of 1305 patients with previously untreated ES-SCLC were included in this study. Data from five trials were collected from the public database Project Data Sphere. Survival analysis and adverse events (AEs) analysis were conducted.

**Results:**

Of the 1305 patients, 800 received the EC regimen whereas 505 received the EP regimen as their front-line treatment. Overall, the median progression-free survival (PFS) and the median overall survival (OS) were 172 and 289 days, respectively. The EP and EC treatment groups did not have significantly different PFS or OS. After adjusting for age, sex, body mass index (BMI) and Eastern Cooperative Oncology Group (ECOG) performance status (PS), the EP regimen was independently associated with better PFS (hazard ratio [HR] = 0.76, 95% CI = 0.63–0.92, *p* = 0.0041) and OS (HR = 0.79, 95% CI = 0.64–0.97, *p* = 0.0220) among patients who were overweight and obese (BMI ≥ 25 kg/m^2^). In the safety analysis, patients who received the EC treatment experienced significantly more grade ≥ 3 AEs (*n* = 599, 74.9%) than those who received the EP treatment (*n* = 337, 66.7%; *p* = 0.002). Furthermore, the EC regimen was associated with a higher risk of grade 3–4 neutropaenia (*p* = 0.001), thrombocytopaenia (*p* < 0.001) and hyponatraemia (*p* = 0.036), whereas the EP regimen was associated with a higher risk of grade 3–4 vomiting (*p* = 0.021).

**Conclusions:**

In summary, this study presented the efficacy and safety of the EC and EP regimens in patients with ES-SCLC in the first-line setting. Patients who are overweight and obese benefit more from the EP regimen than EC regimen. Approaches to define the optimal chemotherapy regimen in different BMI subgroups are needed.

**Supplementary Information:**

The online version contains supplementary material available at 10.1186/s12885-021-09034-6.

## Introduction

Small cell lung cancer (SCLC) is an aggressive disease accounting for approximately 15% of all newly diagnosed lung cancer cases, with an annual global incidence of > 200,000 cases [1, 2]. Despite concurrent chemoradiation and the initial response to platinum-based chemotherapy, the prognosis for this disease remains poor, with a median survival of 20–24 and 10–12 months for patients at the limited and extensive stages, respectively [3].

In terms of systemic treatment for SCLC, most evidence indicates the superiority of platinum-based regimens compared to non-platinum-based ones among de novo patients with extensive-stage small cell lung cancer (ES-SCLC). In the 1970s, cisplatin plus etoposide (EP) demonstrated remarkable activity in patients with SCLC [4]. Since then, the EP regimen has remained the chemotherapy regimen of choice for patients with ES-SCLC. However, despite the benefits of platinum therapy and the wide use of the EP regimen, concerns regarding emetogenicity, nephrotoxicity, ototoxicity and dyselectrolytaemia emerged when using cisplatin, especially among patients with baseline impaired organ function. Although the risk of cisplatin-induced nephrotoxicity could be decreased through hydration, the large volume of this necessary hydration causes clinical inconvenience. Moreover, the prophylactic use of high-dose dexamethasone with cisplatin can impair the immunotherapy benefits when combined with immune checkpoint inhibitors as the novel standard first-line treatment. Therefore, elucidating whether carboplatin can be substituted for cisplatin as the first-line treatment of ES-SCLC is of importance.

In the 1980s, Smith et al. reported that carboplatin plus etoposide (EC) is effective in ES-SCLC, with a response rate of 88% [5]. A randomised phase 3 trial compared the two combinations and found no significant difference in OS, at 12.5 months in the cisplatin arm and 11.8 months in the carboplatin arm. Additionally, patients enrolled in the carboplatin–etoposide arm had better toxicity profiles [6]. Subsequently, Okamoto et al. compared carboplatin (AUC = 5, day 1) with etoposide (80 mg/m^2^, days 1–3) and cisplatin (25 mg/m^2^, days 1–3) with etoposide (80 mg/m^2^, days 1–3), with the two regimens showing equivalent efficacy. Therefore, carboplatin has been indicated as a reasonable substitute for cisplatin in ES-SCLC [7].

Recently, a meta-analysis of 663 individual patient data from four trials compared the efficacy of cisplatin- and carboplatin-based chemotherapy in the first-line treatment of patients with SCLC [8]. Although no differences in efficacy have been identified, different toxicity profiles were confirmed. Notably, the treatment schedules varied among the four trials, including the regimen and dose, which could have resulted in clinical heterogeneity. Moreover, with a third of these patients being in the limited stage, thoracic radiotherapy could have also introduced bias in the results [8]. Therefore, to explore the efficacy and safety difference of EP and EC, we performed the present study to analyse 1305 patients with previously untreated ES-SCLC from five trials using data from the Project Data Sphere.

## Patients and methods

### Patients

The clinical trial inclusion criteria in the present study were as follows: clinical trials involving de novo patients with ES-SCLC, and clinical trials with participants who are receiving carboplatin or cisplatin in combination with etoposide as their antitumor treatment. Trials with systemic antitumor treatment (such as atezolizumab) aside from platinum plus etoposide were excluded. Collectively, five trials were included in the present study: NCT00143455 (phase 3), NCT00363415 (phase 3) [9], NCT00119613 (phase 3) [10], NCT01439568 (phase 2) [11] and NCT02499770 (phase 1b/2) [12]. Using the Project Data Sphere (PDS; www. projectdatasphere.org) platform, de-identified data of patients receiving platinum-etoposide chemotherapy were collected from the five clinical trials for further analysis. Details of these trials are provided in Table S1. Overall, 1427 patients were included in the five trials, and the data of 1305 treatment-naïve patients with ES-SCLC were obtained from the PDS platform. All patients received platinum plus etoposide treatment.

### Clinical variable measures

The retrieved individual data included age at diagnosis, gender, Eastern Cooperative Oncology Group (ECOG) performance status (PS), body mass index (BMI) (underweight: BMI < 18.5 kg/m^2^, normal BMI: 18.5 to < 25 kg/m^2^, overweight: 25 to < 30 kg/m^2^, obese:≥30 kg/m^2^), treatment regimen (EC or EP), serious adverse event (SAE), adverse event (AE), disease status, progression-free survival (PFS), vital status and overall survival (OS).

### Statistical analysis

Pearson’s chi-square and Fisher’s exact tests were used to compare the differences in clinicopathological characteristics. The Kaplan–Meier method was used to calculate survival curves, which were compared using log-rank tests in the univariate analysis. We further identified potential prognostic indicators using Cox proportional hazards regression analysis. A two-sided *p* value < 0.05 was considered significant. Hazard ratios (HRs) and 95% confidence intervals (CIs) were calculated. Fisher’s exact test was used to assess the significance of the association between the chemotherapy regimens (EC versus EP) and grade 1–2 and grade 3–4 AEs. Statistical analyses were conducted using the R version 4.0.3 and SPSS 26.0.

## Results

### Patient characteristics

Of the 1305 patients included, a majority were men (*n* = 928, 68.4%) and the median age was 62 years (range: 28–86 years). ECOG PS ranged from 0 to 2, and only 8.6% of patients had a PS score of 2. A total of 800 patients received the EC regimen, whereas 505 received the EP regimen as their front-line treatment. The median BMI was 25.27 kg/m^2^ (interquartile range [IQR]: 22.22–28.33 kg/m^2^) with 34.6% (*n* = 452) and 17.4% (*n* = 227) of patients in the range of overweight and obese, respectively. The patient characteristics are listed in Table 1.

**Table 1:** Patient characteristics

	EC	EP	Overall
	(*N* = 800)	(*N* = 505)	(*N* = 1305)
Age			
Median [min, max]	63.5 [38.3, 86.2]	60.0 [28.0, 78.0]	62.0 [28.0, 86.2]
Missing data	6 (0.8%)	0 (0%)	6 (0.5%)
Sex			
Female	266 (33.2%)	138 (27.3%)	404 (31.0%)
Male	528 (66.0%)	367 (72.7%)	895 (68.6%)
Missing data	6 (0.8%)	0 (0%)	6 (0.5%)
ECOG PS			
0–1	733 (91.6%)	451 (89.3%)	1184 (90.7%)
2	62 (7.8%)	50 (9.9%)	112 (8.6%)
Missing data	5 (0.6%)	4 (0.8%)	9 (0.7%)
BMI			
Underweight	31 (3.9%)	16 (3.2%)	47 (3.6%)
Normal	347 (43.4%)	228 (45.1%)	575 (44.1%)
Overweight	271 (33.9%)	181 (35.8%)	452 (34.6%)
Obese	148 (18.5%)	79 (15.6%)	227 (17.4%)
Missing data	3 (0.4%)	1 (0.2%)	4 (0.3%)
SAE			
0	493 (61.6%)	215 (42.6%)	708 (54.3%)
1	287 (35.9%)	134 (26.5%)	421 (32.3%)
Missing data	20 (2.5%)	156 (30.9%)	176 (13.5%)
AE			
1	715 (89.4%)	419 (83.0%)	1134 (86.9%)
2	716 (89.5%)	439 (86.9%)	1155 (88.5%)
3	545 (68.1%)	295 (58.4%)	840 (64.4%)
4	231 (28.9%)	148 (29.3%)	379 (29.0%)
5	70 (8.8%)	36 (7.1%)	106 (8.1%)
Missing data	20 (2.5%)	9 (1.8%)	29 (2.2%)
Highest AE^a^			
1	29 (3.6%)	32 (6.3%)	61 (4.7%)
2	152 (19.0%)	127 (25.1%)	279 (21.4%)
3	322 (40.2%)	162 (32.1%)	484 (37.1%)
4	207 (25.9%)	139 (27.5%)	346 (26.5%)
5	70 (8.8%)	36 (7.1%)	106 (8.1%)
Missing data	20 (2.5%)	9 (1.8%)	29 (2.2%)

*Abbreviations*: *EC* etoposide plus carboplatin, *EP* etoposide plus cisplatin, *ECOG* Eastern Cooperative Oncology Group, *PS* performance status, *BMI* body mass index, *SAE* serious adverse event, *AE* adverse event

^a^refers to the highest grade of adverse events in one patient

### Survival analysis

**Fig. 1:**
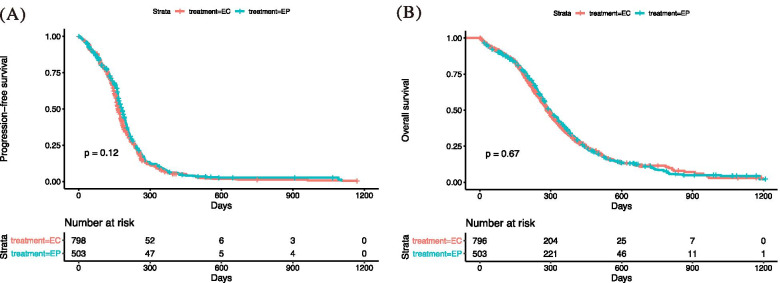
The progression-free survival (**A**) and overall survival (**B**) of patients with extensive-stage small-cell lung cancer according to the treatment groups

After excluding four patients with missing survival data, 1301 patients were included in the survival analysis. Overall, the median PFS was 172 days (95% CI = 167–176) whereas the median OS was 289 days (95% CI = 278–303) (Fig. 1). No significant difference was observed in the EP and EC treatment groups in terms of survival outcomes. The median PFS was 180 and 166 days for patients treated with the EP and EC regimens, respectively (*p* = 0.12), whereas the median OS was 297 and 286 days, respectively (*p* = 0.67). The univariate analysis is presented in Table S2. The multivariate analysis indicated that female patients (HR = 0.81, 95% CI = 0.71–0.93, *p* = 0.0032) had better PFS than their male counterparts. Additionally, being female (HR = 0.72, 95% CI = 0.62–0.83, *p* < 0.0001) and a higher BMI (HR = 0.98, 95% CI = 0.96–0.99, *p* = 0.0013) were independently associated with longer OS, whereas higher ECOG PS (HR = 1.47, 95% CI = 1.31–1.64, *p* < 0.0001) was correlated with worse OS (Table S3).

After adjusting for age, sex, BMI and ECOG PS, the EP regimen was independently associated with better PFS (hazard ratio [HR] = 0.76, 95% CI = 0.63–0.92, *p* = 0.0041) and OS (HR = 0.79, 95% CI = 0.64–0.97, *p* = 0.0220) among patients who were overweight and obese (BMI ≥25 kg/m^2^) (Table 2). However, no significant difference was detected in patients with BMI < 25 kg/m^2^.

**Table 2:** Multivariate analysis of prognostic factors for survival in overweight and obese patient with ES-SCLC

Characteristics	Progression-free survival			Overall survival		
	HR	95%CI	*p* value	HR	95%CI	*p* value
Age	1.00	0.99 - 1.01	0.6869	1.01	1.00 - 1.02	0.1479
Gender	0.86	0.71 - 1.04	0.1270	0.68	0.55 - 0.85	0.0005
BMI	0.99	0.97 - 1.01	0.4396	0.97	0.95 - 1.00	0.0386
ECOG	0.92	0.79 - 1.06	0.2268	1.39	1.20 - 1.62	< 0.0001
EP regimen	0.76	0.63 - 0.92	0.0041	0.79	0.64 - 0.97	0.0220

*Abbreviations*: *HR* hazard ratio, *CI* confidence interval, *ECOG* Eastern Cooperative Oncology Group, *PS* performance status, *BMI* body mass index, *EP* etoposide plus cisplatin

### Safety analysis

Among the 1305 patients (EC: *n* = 800, EP: *n* = 505) included in this study, the AE information of 29 patients was not available. Overall, 770 (96.2%) patients in the EC group and 485 (96.0%) in the EP group have reported the occurrence of grade 1–2 AEs (*p* = 0.44), whereas 583 (72.9%) patients in the EC group and 329 (65.1%) in the EP group have reported the occurrence of grade 3–4 AEs (*p* = 0.005). Patients receiving the EC treatment experienced more grade ≥ 3 AEs (*n* = 599, 74.9%) than those receiving EP (*n* = 337, 66.7%; *p* = 0.002). Moreover, 421 (32.3%) patients from the entire cohort have reported the occurrence of SAEs, among whom 287 (35.9%) were in the EC group and 134 (26.5%) were in the EP group.

In the analysis of grade 1–2 AEs, the top three AEs in the EC group were neutropaenia (33.5%), hypertension (25.8%) and dyspnoea (31.1%), whereas those in the EP group were vomiting (35.8%), neutropaenia (27.9%) and thrombocytopaenia (12.1%). The EC regimen was associated with a higher risk of grade 1–2 haematological toxicities, including anaemia, thrombocytopaenia, hypothyroidism, dyspnoea, infection, hypertension and arrythmia (all *p* < 0.001), hyponatremia (*p* = 0.022) and pneumonia (*p* = 0.013), whereas patients who underwent the EP regimen were more prone to suffer grade 1–2 vomiting, hearing loss and chest pain (all *p* < 0.001). In the analysis of grade 3–4 AEs, the most commonly occurring in the EC group were neutropaenia (41.9%), anaemia (12.2%) and thrombocytopaenia (11.1%), whereas neutropaenia (31.9%), infection (10.3%) and ECOG PS deterioration (9.1%) were the most common in the EP group. The EC regimen was associated with a higher risk of grade 3–4 neutropaenia (*p* = 0.001), thrombocytopaenia (*p* < 0.001) and hyponatremia (*p* = 0.036), whereas the EP regimen was associated with a higher risk of grade 3–4 vomiting (*p* = 0.021). Detailed information on all AEs is presented in Table 3.

**Table 3:** Safety profiles of grade 1–2 and 3–4 adverse events

AEs	EC	EP	Overall	p value
	(N = 800)	(N = 505)	(N = 1305)	
Grade 1–2 AEs				
Neutropaenia	268 (33.5%)	141 (27.9%)	409 (31.3%)	0.106
Anaemia	167 (20.9%)	23 (4.6%)	190 (14.6%)	< 0.001
Thrombocytopaenia	168 (21.0%)	61 (12.1%)	229 (17.5%)	< 0.001
Hyponatremia	24 (3.0%)	4 (0.8%)	28 (2.1%)	0.022
Hyperthyroidism	5 (0.6%)	0 (0%)	5 (0.4%)	0.23
Hypothyroidism	22 (2.8%)	0 (0%)	22 (1.7%)	< 0.001
Vomiting	162 (20.2%)	181 (35.8%)	343 (26.3%)	< 0.001
Dyspnoea	249 (31.1%)	107 (21.2%)	356 (27.3%)	< 0.001
Infection	147 (18.4%)	42 (8.3%)	189 (14.5%)	< 0.001
Pneumonia	31 (3.9%)	6 (1.2%)	37 (2.8%)	0.013
Hearing loss	2 (0.2%)	21 (4.2%)	23 (1.8%)	< 0.001
Hypertension	206 (25.8%)	20 (4.0%)	226 (17.3%)	< 0.001
Chest pain	8 (1.0%)	45 (8.9%)	53 (4.1%)	< 0.001
Arrhythmia	24 (3.0%)	0 (0%)	24 (1.8%)	< 0.001
Thrombosis	18 (2.2%)	2 (0.4%)	20 (1.5%)	0.02
Embolism	4 (0.5%)	0 (0%)	4 (0.3%)	0.288
Grade 3–4 AEs				
Neutropaenia	335 (41.9%)	161 (31.9%)	496 (38.0%)	0.001
Anaemia	98 (12.2%)	41 (8.1%)	139 (10.7%)	0.061
Thrombocytopaenia	89 (11.1%)	26 (5.1%)	115 (8.8%)	< 0.001
Vomiting	11 (1.4%)	19 (3.8%)	30 (2.3%)	0.021
ECOG PS deterioration	46 (5.8%)	46 (9.1%)	92 (7.0%)	0.073
Hyponatremia	35 (4.4%)	9 (1.8%)	44 (3.4%)	0.036
Infection	42 (5.2%)	52 (10.3%)	94 (7.2%)	0.003
Dyspnoea	45 (5.6%)	33 (6.5%)	78 (6.0%)	0.788
Arrhythmia	5 (0.6%)	6 (1.2%)	11 (0.8%)	0.559
Embolism	7 (0.9%)	6 (1.2%)	13 (1.0%)	0.839

*Abbreviations*: *EC* etoposide plus carboplatin, *EP* etoposide plus cisplatin, *AE* adverse event, *ECOG* Eastern Cooperative Oncology Group, *PS* performance status

## Discussion

Recently, the addition of immunotherapy to front-line cytotoxic therapy has further improved patient survival and is recommended as the standard treatment among patients with ES-SCLC. Platinum (cisplatin or carboplatin) plus etoposide remains the backbone chemotherapy regimen for ES-SCLC. The prevalent model of combination therapy further highlights the importance of tolerability and convenience in clinical practice. The present study aimed to investigate the efficacy and safety of EC versus EP in the treatment of patients with ES-SCLC patients. Based on the available data from five clinical trials, we demonstrated that no significant difference exists between the two regimens, as indicated by previous studies, and we presented the prognostic indicators of this population. We also explored the different benefits of both regimens in different subpopulations.

Our findings suggest that female patients had prolonged PFS and OS compared to male patients. In non-small cell lung cancer, the incidence of the driver mutation accounts for the survival difference between male and female patients; in SCLC, this may be explained by the prevalence of smoking. A previous meta-analysis has shown that smoking history was closely related to poorer survival outcomes [13]. Despite smoking status being largely missing in the present analysis, we assumed that the survival difference based on sex could be related to the divergence of smoking status between men and women.

Our pooled analysis also indicated that higher BMI was associated with longer OS. Previous studies have explored the association between BMI and the survival of patients with lung cancer, mainly those with non-small cell lung cancer [14–20], with a majority of studies indicating that higher BMI is associated with improved prognosis. Previous studies have also revealed that patients who are overweight and obese at lung cancer diagnosis have improved OS than those with normal BMI [21, 22]. Similarly, the present study confirmed the prognostic potential of BMI in patients with ES-SCLC receiving platinum-based chemotherapy.

In the present study, we also found that patients who are overweight and obese may derive more benefits from the EP regimen than the EC regimen. The pharmacodynamics of carboplatin is highly dependent on the status of renal function [23] and carboplatin dosing is usually determined by creatinine clearance calculated using the Cockcroft–Gault equation. Notably, bodyweight is one of the variables in the Cockcroft–Gault equation and may lead to overestimation of the carboplatin dose, which may result in more severe AEs, higher incidence of AEs and AE-related mortality [24]. Prospective trials comparing these two regimens in patients with ES-SCLC who are overweight and obese are therefore warranted.

Previously, it has been suggested that there is no difference in efficacy between the EP and EC regimens in the first-line treatment of SCLC [7, 25, 26]. According to the COCIS Meta-Analysis of Individual Patient Data, the median OS for cisplatin and carboplatin was 9.6 and 9.4 months, respectively, which are not significantly different [8]. Regarding the survival of both treatment groups, the data in the present study is comparable to previously reported data. Although the two regimens share similar efficacy, they present significantly different toxicity profiles [25]. As shown in our analysis, the EC regimen was associated with a higher incidence of grade 1–2 and 3–4 AEs compared to the EP regimen, especially in terms of haematologic toxicities. The carboplatin-containing regimen was also correlated with more adverse impacts on the thyroid, which caused higher incidences of grade 1–2 hypothyroidism, and on pulmonary function, which caused higher incidences of grade 1–2 dyspnoea and pneumonia. However, the cisplatin-containing regimen was associated with adverse gastrointestinal effects and neurotoxicity. Recently, immunotherapy has been recommended in combination with chemotherapy in the front-line setting for patients with ES-SCLC [27, 28]. The consideration of the combined toxicity can help us determine the optimal combination for each patient. In the present study, the carboplatin-containing regimen was associated with a higher incidence of thyroid and pulmonary toxicity. Therefore, for patients with co-morbidities that include chronic pulmonary disease and thyroid disease, the incidence of immune-related pneumonitis and thyroid disease should be evaluated when choosing chemotherapy plus immunotherapy.

Despite our large sample size, there are several limitations to this study. First, the retrospective nature and missing detailed information may have introduced difficulties and bias in the analysis. Additionally, treatment-related AEs and AE-related deaths were not analysed owing to the unavailability of the data. Finally, the results of the present study could be influenced by potential confounding owing to the participants’ primary tumour location, metastasis and baseline overall health. Owing to the unbalanced distribution in age and ECOG PS between the two regimens, we had adjusted for these factors in the multivariate analysis to make our analysis as robust as possible. Prospective studies to investigate the dose regimen and intensity during front-line treatment of patients with ES-SCLC in different BMI subgroups are warranted.

## Conclusion

This pooled analysis presented the comparable efficacy and differential safety profile of EC and EP regimens. EP regimen offered more survival benefit in patients with ES-SCLC who are overweight and obese. Further investigations are warranted to define the optimal treatment approach in different BMI subgroups.

## Supplementary Information

**Additional file 1: Table S1**. Trials included in the present study.

**Additional file 2: Table S2**. Univariate analysis of prognostic factors for patient survival.

**Additional file 3: Table S3**. Multivariate analysis of prognostic factors for patient survival.

## Data Availability

Data source: https://data.projectdatasphere.org/projectdatasphere/html/access [five trials namely NCT00143455, NCT00363415, NCT00119613, NCT01439568 and NCT02499770].

## Abbreviations

ES-SCLC: Extensive-stage small cell lung cancer
EP: Cisplatin plus etoposide
EC: Carboplatin plus etoposide
AEs: Adverse events
SAEs: Serious adverse events
OS: Overall survival
PFS: Progression-free survival
PS: Performance status
HR: Hazard ratio
ECOG: Eastern Cooperative Oncology Group
CI: Confidence interval
IQR: Interquartile range
BMI: Body mass index
