# Supplementary material for: Carboplatin versus cisplatin in combination with etoposide in the first-line treatment of small cell lung cancer: a pooled analysis
Source: BMC Cancer. 2021 Dec 7;21:1308. doi: 10.1186/s12885-021-09034-6 (PMC8650295; doi:10.1186/s12885-021-09034-6)
Supplement: Supplementary file 1 — Additional file 1: Table S1. Trials included in the present study. [file 12885_2021_9034_MOESM1_ESM.docx]

**Table S1. Trials included in the present study.**

| Clinical trial number | Patient | Objective | no. of pts | Planned Treatment refimen &dose | Planned cycle | Clinical phase |
| --- | --- | --- | --- | --- | --- | --- |
| NCT00143455 | 18-75 year ES-SCLC ;ECOG PS 0-1 | To compare efficacy of irinotecan hydrochloride with cisplatin versus etoposide with cisplatin as first line  chemotherapy in patients with ED-SCLC. | 243 | ARM B: Etoposide 100 mg/m² , intravenous, day1,2,3  Cisplatin 80 mg/m², intravenous, day1 Both drugs repeated every 3 weeks. | 6 | 3 |
|  |  |  |  |  |  |  |
| NCT00363415 | 18 years old or older ES-SCLC ; ECOG PS 0-2 | Randomized Phase 3 Trial of ALIMTA (Pemetrexed) and Carboplatin versus Etoposide and Carboplatin in ES-SCLC | 455 | etoposide 100 mg/m2, intravenous, days 1-3 x 6 cycles carboplatin AUC=5, intravenous, every 21 days x 6 cycles | 6 | 3 |
| NCT00119613 | 18 years old or older ES-SCLC ; ECOG PS 0-2 | A Study of Subjects With Previously Untreated ES-SCLC Treated With Platinum Plus Etoposide Chemotherapy With or Without Darbepoetin Alfa | 600 | etoposide 80-120 mg/m2, intravenous, days 1-3；carboplatin AUC=5 – 6/cisplatin 60-100 mg/m2 /cycle will be  given over 1-3 days | 6 | 3 |
| NCT01439568 | 18 years old or older ES-SCLC; ECOG PS 0-2 | A Study of LY2510924 in Participants With ES-SCLC | 92 | etoposide 100 mg/m2, intravenous, days 1-3 x 6 cycles carboplatin AUC=5, intravenous, every 21 days x 6 cycles | 6 | 2 |
| NCT02499770 | 18 years old or older ES-SCLC; ECOG PS 0-2 | A Study of G1T28 compared with placebo in Patients With ES-SCLC Receiving Etoposide and Carboplatin | 37 | etoposide 100 mg/m2, intravenous, days 1-3 x 6 cycles carboplatin AUC=5, intravenous, every 21 days | median 5(range 1-8) | 1b/2 |

Abbreviations: ES-SCLC, extensive-stage small cell lung cancer; ECOG, Eastern Cooperative Oncology Group; PS, performance status; AUC, area under the curve.
