# Supplementary material for: Carboplatin versus cisplatin in combination with etoposide in the first-line treatment of small cell lung cancer: a pooled analysis
Source: BMC Cancer. 2021 Dec 7;21:1308. doi: 10.1186/s12885-021-09034-6 (PMC8650295; doi:10.1186/s12885-021-09034-6)
Supplement: Supplementary file 2 — Additional file 2: Table S2. Univariate analysis of prognostic factors for patient survival. [file 12885_2021_9034_MOESM2_ESM.docx]

**Table S2. Univariate analysis of prognostic factors for patient survival.**

| Characteristics | Progression-free survival | | | Overall survival | | |
| --- | --- | --- | --- | --- | --- | --- |
|  | HR | 95%CI | *p* value | HR | 95%CI | *p* value |
| Age | 1.00 | 0.99 - 1.01 | 0.594 | 1.01 | 1.00 - 1.02 | 0.0188 |
| Gender | 0.82 | 0.71 - 0.94 | 0.004 | 0.74 | 0.64 - 0.86 | <0.0001 |
| BMI | 1.00 | 0.98 - 1.01 | 0.49 | 0.98 | 0.96 - 0.99 | 0.0003 |
| ECOG | 1.03 | 0.93 - 1.14 | 0.557 | 1.44 | 1.30 - 1.61 | <0.0001 |
| EP regimen | 0.90 | 0.80 - 1.03 | 0.123 | 0.97 | 0.85 - 1.11 | 0.671 |

Abbreviations: HR, hazard ratio; CI, confidence interval; ECOG, Eastern Cooperative Oncology Group; PS, performance status; BMI, body mass index; EP, etoposide plus cisplatin.
