# Supplementary material for: Carboplatin versus cisplatin in combination with etoposide in the first-line treatment of small cell lung cancer: a pooled analysis
Source: BMC Cancer. 2021 Dec 7;21:1308. doi: 10.1186/s12885-021-09034-6 (PMC8650295; doi:10.1186/s12885-021-09034-6)
Supplement: Supplementary file 3 — Additional file 3: Table S3. Multivariate analysis of prognostic factors for patient survival. [file 12885_2021_9034_MOESM3_ESM.docx]

**Table S3. Multivariate analysis of prognostic factors for patient survival.**

| Characteristics | Progression-free survival | | | Overall survival | | |
| --- | --- | --- | --- | --- | --- | --- |
|  | HR | 95% CI | p value | HR | 95% CI | p value |
| Age | 1.00 | 0.99–1.01 | 0.9489 | 1.01 | 1.00–1.01 | 0.2060 |
| Sex | 0.81 | 0.71–0.93 | 0.0032 | 0.72 | 0.62–0.83 | <0.0001 |
| BMI | 1.00 | 0.99–1.01 | 0.6389 | 0.98 | 0.96–0.99 | 0.0013 |
| ECOG PS | 1.06 | 0.95–1.18 | 0.2926 | 1.47 | 1.31–1.64 | <0.0001 |
| EP regimen | 0.88 | 0.77–1.01 | 0.0699 | 0.87 | 0.76–1.01 | 0.0674 |

Abbreviations: HR, hazard ratio; CI, confidence interval; ECOG PS, Eastern Cooperative Oncology Group performance status, BMI, body mass index; EP, etoposide plus cisplatin.
